# Multienvironment genomic variance decomposition analysis of open-pollinated Interior spruce (*Picea glauca* x *engelmannii*)

**DOI:** 10.1007/s11032-018-0784-3

**Published:** 2018-02-15

**Authors:** Omnia Gamal El-Dien, Blaise Ratcliffe, Jaroslav Klápště, Ilga Porth, Charles Chen, Yousry A. El-Kassaby

**Affiliations:** 1 Department of Forest and Conservation Sciences, Faculty of Forestry, University of British Columbia, Vancouver, BC V6T 1Z4 Canada; 2 Pharmacognosy Department, Faculty of Pharmacy, Alexandria University, Alexandria, Egypt; 3 Department of Genetics and Physiology of Forest Trees, Faculty of Forestry and Wood Sciences, Czech University of Life Sciences Prague, Kamycka 129, 165 21 Prague 6, Czech Republic; 4 Present Address: Scion (New Zealand Forest Research Institute Ltd.), 49 Sala Street, Whakarewarewa, Rotorua, 3046 New Zealand; 5 Present Address: Départment des Sciences du Bois et de la Forêt, Faculté de Foresterie, de Géographie et Géomatique, Université Laval, Quebec City, QC G1V 0A6 Canada; 6 Department of Biochemistry and Molecular Biology, Oklahoma State University, Stillwater, OK 74078-3035 USA

**Keywords:** Open-pollinated families, Interior spruce, Multienvironment, Genetic variance decomposition, Pedigree- and marker-based relationships

## Abstract

The advantages of open-pollinated (OP) family testing over controlled crossing (i.e., structured pedigree) are the potential to screen and rank a large number of parents and offspring with minimal cost and efforts; however, the method produces inflated genetic parameters as the actual sibling relatedness within OP families rarely meets the half-sib relatedness assumption. Here, we demonstrate the unsurpassed utility of OP testing after shifting the analytical mode from pedigree- (ABLUP) to genomic-based (GBLUP) relationship using phenotypic tree height (HT) and wood density (WD) and genotypic (30k SNPs) data for 1126 38-year-old Interior spruce (*Picea glauca* (Moench) Voss x *P. engelmannii* Parry ex Engelm.) trees, representing 25 OP families, growing on three sites in Interior British Columbia, Canada. The use of the genomic realized relationship permitted genetic variance decomposition to additive, dominance, and epistatic genetic variances, and their interactions with the environment, producing more accurate narrow-sense heritability and breeding value estimates as compared to the pedigree-based counterpart. The impact of retaining (random folding) vs. removing (family folding) genetic similarity between the training and validation populations on the predictive accuracy of genomic selection was illustrated and highlighted the former caveats and latter advantages. Moreover, GBLUP models allowed breeding value prediction for individuals from families that were not included in the developed models, which was not possible with the ABLUP. Response to selection differences between the ABLUP and GBLUP models indicated the presence of systematic genetic gain overestimation of 35 and 63% for HT and WD, respectively, mainly caused by the inflated estimates of additive genetic variance and individuals’ breeding values given by the ABLUP models. Extending the OP genomic-based models from single to multisite made the analysis applicable to existing OP testing programs.

## Introduction

Traditional quantitative genetics analyses are mainly pedigree dependent utilizing the genealogical relationships among individuals for genetic parameter estimation (i.e., the average numerator relationship matrix (***A***-matrix; Wright 1922)). These methods were effective as evidenced by the gains attained for a substantial number of plant and animal genetic improvement programs (Allard 1999; Lush 2013). However, this paradigm is changing with the availability of dense single nucleotide polymorphism (SNP) panels through whole-genome sequencing (Bentley 2006) and various high-throughput next-generation sequencing (NGS) technologies (Schuster 2008). Dense sequencing data permit the accurate determination of the actual fraction of alleles shared between individuals, related or otherwise, and the estimation of their genomic pairwise-realized relationship (Santure et al. 2010). The resulting genomic relationship between any pair of individuals is more accurate than their expected pedigree-based relationship as genomic data capture their known contemporary and unknown historic pedigree (Powell et al. 2010). When the genomic pairwise additive relationship is estimated for a group of individuals, the outcome is known as the realized additive genomic relationship matrix (***G***-matrix) which can be used as a substitute to the ***A***-matrix in quantitative genetics analyses (VanRaden 2008). Also, SNP data can be used to construct all types of relationship matrices such as dominance and epistasis genomic relationship matrices regardless of the mating design (Wang et al. 2014). The advantage of the genomic- over pedigree-based relationship is the ability of the former to adjust for the Mendelian sampling term, while the latter ignores the existing variation among single half- or full-sib family members and treats them equally; thus, the ***G***-matrix provides more accurate genetic co-variances among relatives (Visscher et al. 2006; Hill and Weir 2011). Additionally, the genomic-based relationship is capable of detecting inbreeding and hidden relatedness among members of a specific family. The use of the genomic relationship matrix in disentangling full-sib families’ genetic variance components (i.e., additive and nonadditive) has been thoroughly investigated theoretically (Denis and Bouvet 2013; Vitezica et al. 2013; Motohide and Satoh 2014; Azevedo et al. 2015; de Almeida Filho et al. 2016) and empirically (Vitezica et al. 2013; Zapata-Valenzuela et al. 2013; Motohide and Satoh 2014; Muñoz et al. 2014; Kumar et al. 2015; Bouvet et al. 2016; de Almeida Filho et al. 2016); however, with the exception of Gamal El-Dien et al. (2016), a study that was based on open-pollinated (OP) families growing on a single site, OP families have not received much attention.

The success of forest trees recurrent selection programs is dependent on the identification of superior individuals for their inclusion in subsequent breeding cycles and/or seed production populations (i.e., seed orchards) (El-Kassaby 1995). Testing is commonly done through creating a structured pedigree using mating designs, which is followed by classical quantitative genetic evaluation for determining parents’ and offspring’s genetic merit (Namkoong et al. 1988). The major drawback of OP families is the unknown nature of their paternal contribution (i.e., incomplete pedigree), even though they have been commonly used in testing as they offer multiple advantages over their “structured pedigree” counterparts (Burdon and Shelbourne 1971). These include the following: (1) it is fast and inexpensive as the breeding phase is bypassed (i.e., no crosses), (2) it permits screening large numbers of parents (seed donors) with minimal efforts, (3) it provides better genetic sampling as each seed donor acts as a conduit for many pollen donors, and (4) it offers greater selection differential through increased candidate testing. However, it should be stated that the OP families’ pedigree-based additive genetic variance estimates are always upwardly bias as the assumption of true half-siblings cannot be verified/fulfilled (Namkoong 1966; Squillace 1974; Askew and El-Kassaby 1994).

In a previous study, Gamal El-Dien et al. (2016) utilized the genomic relationship to estimate height- and wood density-related (proxies for fitness and productivity) genetic variances for 214 white spruce OP families growing on one site in Québec, Canada, and successfully partitioned the genetic variance into its different components, namely, the additive, dominance, and epistatic variances, and demonstrated the presence of a systematic pedigree-based additive genetic variance bias. In this respect, the use of the genomic relationship also permitted estimating both dominance and epistatic genetic variances from a testing experiment that does not lend itself to the estimation of these genetic components.

Here, using 25 Interior spruce OP families grown in a replicated block design over three sites in British Columbia, Canada, we compared the genetic variance estimates generated from the average numerator relationship ***A***-matrix (the expected relationships) and the realized genomic relationship ***G***-matrix (the observed relationships). More specifically, we evaluated the genomic selection predictive ability after removing genetic relatedness between the training and validation populations during the cross-validation process and determined the response to selection differences between the expected and realized relationships for the studied attributes. This OP progeny test permitted extending the single-site analysis to a more generalized multiple-site model that partitioned the genetic variance components into additive and nonadditive effects as well as accounting for and determining the extent of genotype × environment interaction, thus allowing covering a more complex genetic structure.

## Materials and methods

### Progeny test and phenotypic and genotypic data

Interior spruce is a complex of white spruce (*Picea glauca* (Moench) Voss), Engelmann spruce (*Picea engelmannii* Parry), and their natural hybrids, and because of their similar growing habitat and silvicultural requirements, they are often collectively treated as one complex species (Sutton et al. 1991). A total of 1126 38-year-old Interior spruce trees, representing 25 OP families, growing on three progeny test sites in the Interior of British Columbia, Canada, were phenotyped for total tree height (HT) and wood density (WD). The field trials were established by the British Columbia Ministry of Forests, Lands and Natural Resource and are located in Aleza Lake (lat. 54° 03′ 15.7″ N, long. 122° 06′ 35.4″ W, elev. 700 mas), Prince George Tree Improvement Station (lat. 53° 46′ 17.9″ N, long. 122° 43′ 07.6″ W, elev. 610 mas), and Quesnel (lat. 52° 59′ 27.2″ N, long. 122° 12′ 30.6″ W, elev. 915 mas) and planted in a complete randomized block design with multiple tree-row-plots within each block (see Kiss (1991) for details). The sampled trees/sites are part of a larger test with 197 OP families with an average family size of 374 trees. From each site, four blocks (replications) were sampled and HT (in meters) was measured using an ultrasonic clinometer Vertex™ III (Haglöf^®^, Sweden); WD (g·cm^−3^) was determined from bark-to-bark wood cores using X-ray scanning (QTRS-01X Tree Ring Scanner, Quintek Measurement Systems Inc., USA); the cores were extracted from each tree at breast height in the north-south direction by 5-mm increment borers.

Genotyping-by-sequencing (GBS: Elshire et al. (2011)) was the genotyping platform used. For complete details related to DNA extraction, specific sequencing protocol, and SNP detection pipeline, see Chen et al. (2013). The SNP data used for estimating the realized genomic relationship matrix were those published previously (Gamal El-Dien et al. 2015; Ratcliffe et al. 2015); in brief, SNP filtering consisted of constraining individual “missingness” to the best 1000 of the 1126 genotyped individuals, resulting in an average of 40 genotyped individuals (range was 32 to 45) across the 25 families. Subsequently, SNPs with less than 30% missing data were retained, and missing information was imputed using an expectation maximizing (EM) algorithm (Dempster et al. 1977), resulting in a total of ~ 30,000 SNP markers which were used to infer the genomic relationships (i.e., the SNP data were filtered for a missing data threshold of 30% followed by EM algorithm imputation resulting in 1000 individuals with ~ 30,000 SNPs).

### Relationship matrices and genetic models

The additive relationship matrix was estimated as follows:

1 $$ {\boldsymbol{G}}_{\mathbf{add}}=\frac{{\boldsymbol{ZZ}}^{\prime }}{2\sum {p}_i\left(1-{p}_i\right)} $$

where ***Z*** is the rescaled genotype matrix following ***M*** − ***P***; ***M*** is the genotype matrix containing genotypes coded as 0, 1, and 2 according to the number of alternative alleles; and ***P*** is the vector of twice the allelic frequency *p* (VanRaden 2008). The dominance genetic variance was fitted by including the marker-based dominance relationship matrix following:

2 $$ {\boldsymbol{G}}_{\mathbf{dom}}=\frac{{\boldsymbol{WW}}^{\prime }}{{\left(2 pq\right)}^2} $$

where ***W*** is the matrix containing − 2*q*^2^ for the alternative homozygote, 2*pq* for the heterozygote, and − 2*p*^2^ for the reference allele homozygote (Vitezica et al. 2013). Similarly, epistatic variance was fitted by including several relationship matrices capturing first-order additive × additive, dominance × dominance, and additive × dominance interaction. The relationship matrices were constructed as the Hadamard product of the relationship matrices defined above: ***G***_**add**_#***G***_**add**_, ***G***_**dom**_#***G***_**dom**_, and ***G***_**add**_#***G***_**dom**_ (Su et al. 2012; Muñoz et al. 2014).

The variance components from the pedigree-based analysis (ABLUP) were obtained by solving the mixed models following:

3 $$ \boldsymbol{y}=\boldsymbol{X}\boldsymbol{\beta } +{\boldsymbol{Z}}_{\mathbf{1}}\boldsymbol{a}+{\boldsymbol{Z}}_{\mathbf{2}}\boldsymbol{a}\boldsymbol{xe}+{\boldsymbol{Z}}_{\mathbf{3}}\boldsymbol{r}\left(\boldsymbol{s}\right)+\boldsymbol{e} $$

where ***y*** is the vector of standardized phenotype values to cope with the possible heterogeneity of variance between environments; ***β*** is the vector of fixed effects (overall mean and site); ***a*** is the vector of random additive genetic effects following ***a*** ~ *N*(0, ***A***${\sigma}_a^2$), where ***A*** is the average numerator relationship matrix and ${\sigma}_a^2$ is the additive genetic variance; ***axe*** is the vector of random additive × environment (sites) interaction effects following ***axe*** ~ *N*(0, ***I***${\sigma}_{axe}^2$), where ***I*** is the identity matrix and ${\sigma}_{axe}^2$ is the additive × environment interaction variance; ***r***(***s***) is the vector of random block (replication) nested within the site effect following ***r***(***s***) ~ *N*(0, ***I***${\sigma}_{r(s)}^2$), where ${\sigma}_{r(s)}^2$ is the replication nested within the site variance; ***e*** represents a vector of the random residual effects following ***e*** ~ *N*(0, ***I***${\sigma}_e^2$), where ${\sigma}_e^2$ is the residual error variance; and ***X*** and ***Z***’s are incidence matrices relating fixed and random effects to measurements in the vector ***y***. The variance components from the analysis using the marker-based additive relationship matrix (GBLUP-A) were obtained from the model described above, but the average numerator relationship matrix ***A*** is substituted by the marker-based relationship matrix ***G***_**add**_. The extended model for the dominance term (GBLUP-AD) is performed as follows:

4 $$ \boldsymbol{y}=\boldsymbol{X}\boldsymbol{\beta } +{\boldsymbol{Z}}_{\mathbf{1}}\boldsymbol{a}+{\boldsymbol{Z}}_{\mathbf{4}}\boldsymbol{d}+{\boldsymbol{Z}}_{\mathbf{2}}\boldsymbol{a}\boldsymbol{xe}+{\boldsymbol{Z}}_{\mathbf{5}}\boldsymbol{d}\boldsymbol{xe}+{\boldsymbol{Z}}_{\mathbf{3}}\boldsymbol{r}\left(\boldsymbol{s}\right)+\boldsymbol{e} $$

where ***d*** is the vector of the random dominance effect following ***d*** ~ *N*(0, ***G***_**dom**_${\sigma}_d^2$) with ${\sigma}_d^2$ the dominance variance and ***dxe*** the random vector of dominance × environment interaction effects following ***dxe*** ~ *N*(0, ***I***${\sigma}_{dxe}^2$), where ${\sigma}_{dxe}^2$ is the dominance × environment interaction variance. Additional model extension for epistatic terms (GBLUP-ADE) is performed as follows:

5 $$ \boldsymbol{y}=\boldsymbol{X}\boldsymbol{\beta } +{\boldsymbol{Z}}_{\mathbf{1}}\boldsymbol{a}+{\boldsymbol{Z}}_{\mathbf{4}}\boldsymbol{d}+{\boldsymbol{Z}}_{\mathbf{6}}\boldsymbol{a}\boldsymbol{xa}+{\boldsymbol{Z}}_{\mathbf{7}}\boldsymbol{d}\boldsymbol{xd}+{\boldsymbol{Z}}_{\mathbf{8}}\boldsymbol{a}\boldsymbol{xd}+{\boldsymbol{Z}}_{\mathbf{2}}\boldsymbol{a}\boldsymbol{xe}+{\boldsymbol{Z}}_{\mathbf{5}}\boldsymbol{d}\boldsymbol{xe}+{\boldsymbol{Z}}_{\mathbf{3}}\boldsymbol{r}\left(\boldsymbol{s}\right)+\boldsymbol{e} $$

where ***axa*** is the vector of random additive × additive epistatic interaction effects following ***axa*** ~ *N*(0, ***G***_**add#add**_${\sigma}_{axa}^2$), where ${\sigma}_{axa}^2$ is the additive × additive epistatic interaction variance; ***dxd*** is the vector of random dominance × dominance epistatic interaction effects following ***dxd*** ~ *N*(0, ***G***_**dom#dom**_${\sigma}_{dxd}^2$), where ${\sigma}_{dxd}^2$ is dominance × dominance epistatic interaction variance; and ***axd*** is the vector of random additive × dominance epistatic interaction effects following ***axd*** ~ *N*(0, ***G***_**add#dom**_${\sigma}_{axd}^2$), where ${\sigma}_{axd}^2$ is the additive × dominance epistatic interaction variance.

The narrow-sense heritability estimate was estimated as ${\widehat{h}}^2={\widehat{\sigma}}_a^2/{\widehat{\sigma}}_p^2$, where ${\widehat{\sigma}}_a^2$ represents the estimate of the additive variance and ${\widehat{\sigma}}_p^2$ equals ${\widehat{\sigma}}_e^2$ in addition to the other variance component estimates such as additive, dominance, additive × additive, additive × dominance, dominance × dominance, additive × environment, and dominance × environment interactions following that of the ABLUP and GBLUP (termed GBLUP-A, GBLUP-AD, and GBLUP-ADE, respectively) models, respectively (Table 1). Where possible, the broad-sense heritability was also estimated as ${\widehat{H}}^2={\widehat{\sigma}}_{\mathrm{G}}^2/{\widehat{\sigma}}_p^2$, where ${\widehat{\sigma}}_{\mathrm{G}}^2\ \mathrm{represents}\ \mathrm{the}\ \mathrm{sum}\ \mathrm{of}\ \mathrm{all}\ \mathrm{genetic}\ \mathrm{effects}.$ The estimations of the variance components and their standard errors were performed using ASReml-R v.3. software (Butler et al. 2009, while the marker-based relationship matrices construction and models’ cross-validations were done in R (R Core Team 2015). Additionally, the rank order of breeding values (BVs) for the top 50 performing individuals was compared between ABLUP and GBLUP-AD and GBLUP-ADE for HT and WD, respectively.

**Table 1:** Estimates of genetic variance components (source of variation (S.O.V.) and their standard errors (SE)) for height (HT) and wood density (WD) across the four genetic models

Trait	S.O.V.	ABLUP		GBLUP-A		GBLUP-AD		GBLUP-ADE	
		Value (SE)	%	Value (SE)	%	Value (SE)	%	Value (SE)	%
HT	${\sigma}_{r(s)}^2$	0.02 (0.01)	1.63	0.02 (0.01)	1.76	0.02 (0.01)	2.07	0.02 (0.01)	2.07
	${\sigma}_a^2$	0.31 (0.15)	30.28	0.21 (0.08)	23.70	0.19 (0.08)	24.38	0.19 (0.08)	24.38
	${\sigma}_d^2$	N/A		N/A		0.15 (0.14)	19.46	0.15 (0.14)	19.46
	${\sigma}_{axa}^2$	N/A		N/A		N/A		0.00 (0.00)	0.00
	${\sigma}_{dxd}^2$	N/A		N/A		N/A		0.00 (0.00)	0.00
	${\sigma}_{axd}^2$	N/A		N/A		N/A		0.00 (0.00)	0.00
	${\sigma}_{axe}^2$	0.35 (0.13)	34.73	0.22 (0.08)	24.68	0.22 (0.09)	28.14	0.22 (0.09)	28.14
	${\sigma}_{dxe}^2$	N/A		N/A		0.03 (0.19)	4.54	0.03 (0.19)	4.54
	${\sigma}_e^2$	0.34 (0.14)	33.37	0.45 (0.09)	49.86	0.16 (0.27)	21.41	0.16 (0.27)	21.41
	*h* ^2^	0.31 (0.15)		0.24 (0.09)		0.25 (0.12)		0.25 (0.12)	
	*H* ^2^	N/A		N/A		0.45 (0.23)		0.45 (0.23)	
	*R* ^2^	66.63		50.14		78.59		78.59	
WD	${\sigma}_{r(s)}^2$	0.07 (0.04)	6.91	0.07 (0.04)	7.71	0.07 (0.04)	8.21	0.07 (0.04)	8.62
	${\sigma}_a^2$	0.37 (0.15)	36.66	0.22 (0.07)	23.89	0.22 (0.07)	25.33	0.18 (0.08)	21.64
	${\sigma}_d^2$	N/A		N/A		0.00 (0.14)	0.29	0.02 (0.14)	2.76
	${\sigma}_{axa}^2$	N/A		N/A		N/A		0.16 (0.18)	19.26
	${\sigma}_{dxd}^2$	N/A		N/A		N/A		0.00 (0.00)	0.00
	${\sigma}_{axd}^2$	N/A		N/A		N/A		0.00 (0.00)	0.00
	${\sigma}_{axe}^2$	0.17 (0.09)	16.98	0.12 (0.07)	13.30	0.11 (0.08)	12.47	0.12 (0.08)	13.75
	${\sigma}_{dxe}^2$	N/A		N/A		0.07 (0.23)	7.86	0.01 (0.23)	1.32
	${\sigma}_e^2$	0.40 (0.13)	39.45	0.52 (0.08)	55.10	0.41 (0.29)	45.84	0.28 (0.32)	32.66
	*h* ^2^	0.39 (0.16)		0.26 (0.09)		0.28 (0.13)		0.24 (0.12)	
	*H* ^2^	N/A		N/A		0.28 (0.18)		0.48 (0.32)	
	*R* ^2^	60.55		44.90		54.16		67.34	

### Comparison and cross-validation of models

Finally, to compare the relative quality of the goodness-of-fit for the said models, the variance explained by each model (*R*^2^) was used (Nakagawa and Schielzeth 2013), that is the summary statistics for the goodness-of-fit of the linear mixed-effects models (LMM) and the fitted line plot (graph of predicted *ŷ* vs. *y* values), while the standard error (SE) of the predictions (SEPs) of the BVs was used to assess the precision of the BVs.

The predictability (i.e., the Pearson product-moment correlation between phenotypes and the predicted BVs from cross-validation (PBV-CV)) and the prediction accuracy (i.e., the Pearson product-moment correlation between the estimated BVs from full data (EBV-all) and predicted BVs from cross-validation (PBV-CV)) for the four models were estimated using 10-fold CV and five replicates. To assess the role of relatedness between the training and validation populations on the genomic selection predictive accuracy, two folding scenarios were used, namely, random (retained relatedness as individuals were removed during the CV process while their families remained) and family (removed relatedness as entire families were absent during the CV process) folding. In each replicate, the data was divided into 10-folds according to the used folding scenario, 9-folds was assigned as the training population, while the last fold was used as the validation population to estimate PBV-CV. The five replicates were used to estimate the SE of the correlation. Model pairwise prediction accuracy was also estimated between the four models in order to evaluate the ability of predicting each other. In this case, accuracy was estimated as the Pearson product-moment correlation between EBV-all of one model and PBV-CV of the other model (see above).

## Results

### Genetic variance components and heritability estimates

Replications within-site variance components were consistent across the four models and accounted in each case for a relatively small variance component for both height (HT 1.63–2.07%) and wood density (WD 6.91–8.62%) (Table 1). The main difference between the ABLUP and GBLUP-A was the substantial decrease in the additive variance and additive × environment interaction (Table 1). The additive genetic variances obtained from GBLUP-A were 68 and 59% of the ABLUP additive genetic variance for HT and WD estimates, respectively (Table 1). This decrease in the additive genetic variance apportionment subsequently decreased the additive × environment interaction (34.73 vs. 24.68% and 16.98 vs. 13.30%, for height HT and WD, respectively) and increased the residual term (33.37 vs. 49.86% and 39.45 vs. 55.10%, for height HT and WD, respectively), resulting in reduced narrow-sense heritability estimates (0.31 vs. 0.24 and 0.39 vs. 0.26, for height HT and WD, respectively) (Table 1). Broad-sense heritabilities could not be estimated for the ABLUP and GBLUP-A as dominance and epistatic variances could not be estimated; however, GBLUP-AD and GBLUP-ADE produced similar values for height (0.45) and drastically higher estimate for WD (0.28 vs. 0.48, see below for explanation) (Table 1).

The GBLUP-AD analysis produced surprising results for HT as the dominance variance component was significant and accounted for 19.46% of the total variance, while it was nonsignificant for WD (0.29%) (Table 1). It is noteworthy to mention that the dominance variance estimates affected neither the additive genetic variances nor the heritability estimates and that their appearance is mostly reflected in the reduction of the residual term estimates (i.e., the dominance variances were confounded in the residual terms) (Table 1).

The GBLUP-ADE produced exactly the same results as GBLUP-AD for HT, indicating the absence of first-order interactions, while WD produced substantial additive × additive interaction accounting for 19.26% of the total variance (Table 1). The appearance of additive × additive variance for WD reduced the residual term (45.84 vs. 32.66%) as well as the additive term (25.33 vs. 21.64%) for GBLUP-AD and GBLUP-ADE, respectively, further changing the WD heritability estimates (narrow-sense: from 0.28 to 0.24 and broad-sense: from 0.28 to 0.48) (Table 1).

In the ABLUP model, the genotype × environmental interaction (G × E) can only be expressed through the terms “additive variance × environment,” and these were the first and third highest variance components, accounting for 34.73 and 16.98% of the total variance for HT and WD, respectively (Table 1). Under the GBLUP model, the additive variance × environment interactions magnitude showed slight rank change for the studied traits (HT: shifting from first to second; WD: retained the same rank); however, both showed appreciable percentage reduction (HT: from 34.73 to 24.68%; WD: from 16.98 to 13.30%) (Table 1). Changing the model from ABLUP to GBLUP produced consistent redistribution of the variance components across HT and WD, notably the reduction in additive genetic variance and additive × environment interaction terms, resulting in residual terms increase (HT: from 33.37 to 49.86; WD: from 39.45 to 55.10%) (Table 1). Dominance variance × environment interaction can only be observed in both GBLUP-AD and GBULP-ADE models where dominance variance can be estimated (Table 1). While HT and WD displayed different dominance genetic variance magnitudes (see above), both traits displayed small dominance variance × environment interactions (HT, 4.54% for GBLUP-AD and GBLUP-ADE; WD, 7.86 and 1.32% for GBLUP-AD and GBLUP-ADE, respectively) (Table 1). Considering the G × E interaction (i.e., additive and dominance variances) in the genetic variance, decomposition resulted in the production of more reliable heritability estimates and this is supported by the model fit results (see below).

### Comparison and cross-validation of models

**Fig. 1:**
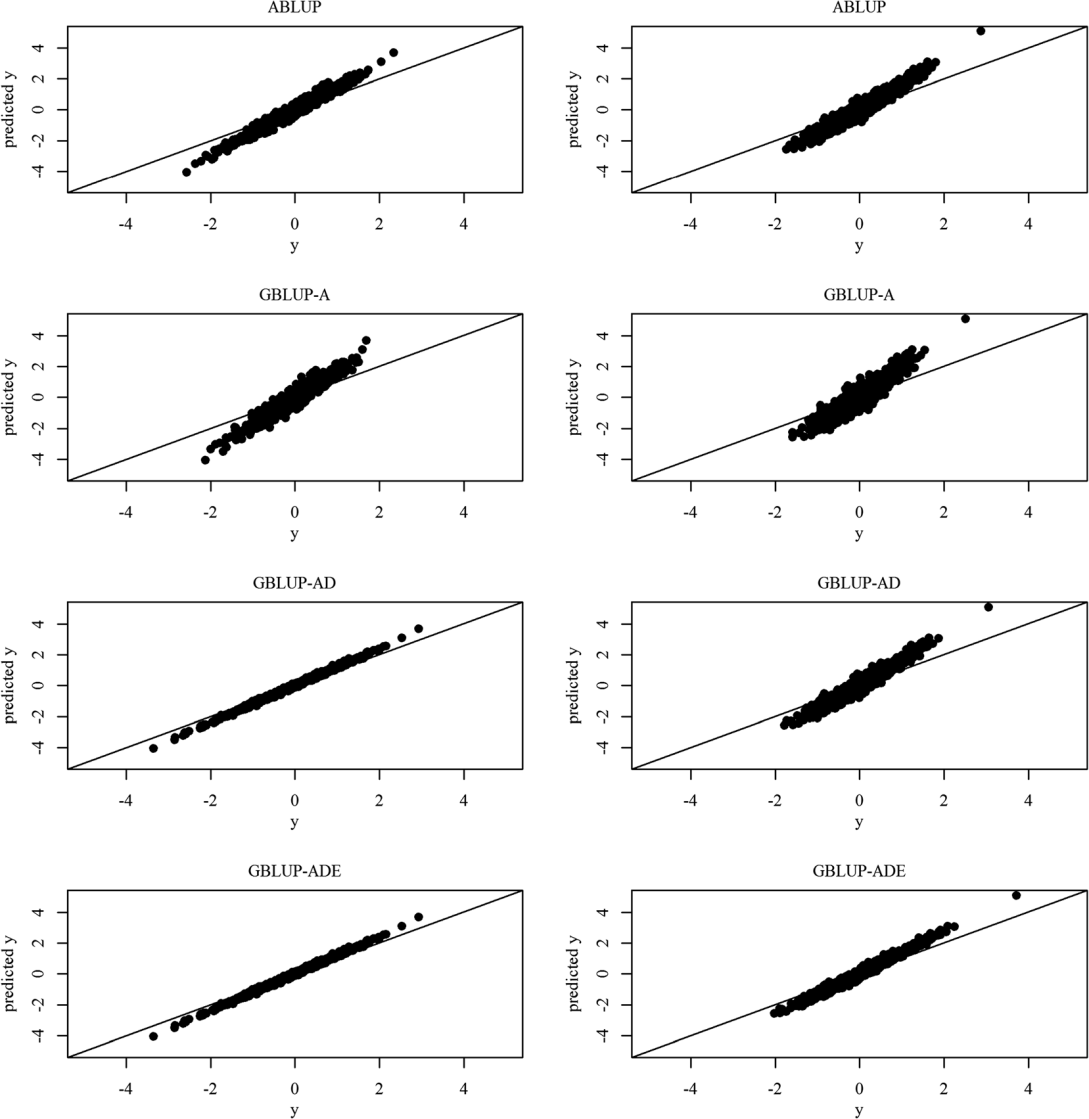
Height (left) and wood density (right) fitted line plot (predicted *ŷ* vs. observed *y* values) for the four models

We used two methods for model comparison, namely, the variance explained by the model (*R*^2^) and the fitted line plots (represented by the graph of predicted values *ŷ* vs. observed values *y*). Moving from ABLUP to the GBLUP-A was characterized by the lack of improvement for the two model comparison methods (Table 1 and Fig. 1). However, this result is not surprising, as the ABLUP models were inaccurate due to the observed inflated additive genetic variance which in turn makes the total variance explained by the model inflated, too. The *R*^2^ method showed reduced values between ABLUP and GBLUP-A (66.63 vs. 50.14 and 60.55 vs. 44.90, for HT and WD, respectively) (Table 1). Comparing GBLUP-A with GBLUP-AD generally showed improvement, which was more pronounced for HT (50.14 vs. 78.59) than for WD (44.90 vs. 54.16) due to the observed dominance variance (Table 1). The *R*^2^ values for HT did not change between GBLUP-AD and GBLUP-ADE due to the lack of epistatic genetic variances, indicating that GBLUP-AD is the best (and sufficient) model for HT (Table 1). WD, on the other hand, produced substantial *R*^2^ value improvement (54.16 vs. 67.34), reflecting the presence of significant additive × additive genetic variances and indicating that GBLUP-ADE is the best model for WD. The *R*^2^ differences between HT and WD reflect the different genetic architecture of the two traits (Table 1). These results collectively indicate that the genomic-based models are superior to the pedigree-based model.

The fitted line plot comparisons (shown in Fig. 1) reflected the conclusions based on *R*^2^, while the differences between the ABLUP and GBLUP-A models for HT and WD showed worse fitting, supporting the notion that the ABLUP models harbor inflated additive genetic variance. Similarly, the plots show that the GBLUP-AD and GBLUP-ADE are the best fit for HT and WD, respectively, and this is illustrated by the points’ distribution and their closeness to the 45° reference lines (Fig. 1).

**Fig. 2:**
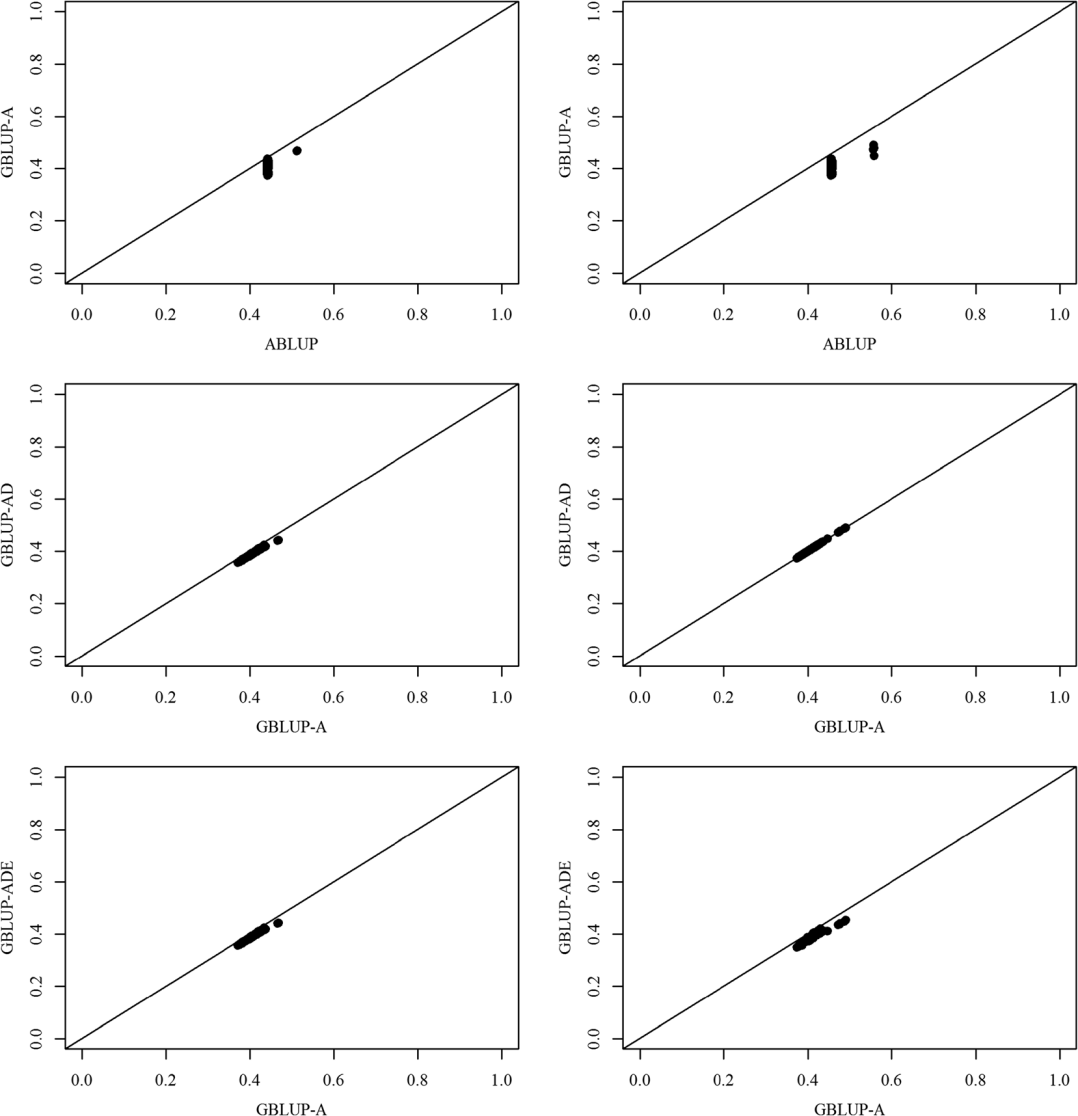
Height (left) and wood density (right) standard error for the predictions of 38-year-old Interior spruce breeding value comparisons for GBLUP-A vs. ABLUP, GBLUP-AD vs. GBLUP-A, and GBLUP-ADE vs. GBLUP-A

Comparing BVs’ precision using the SEPs between the ABLUP and GBLUP-A models indicated that the SEPs of HT and WD were universally smaller for GBLUP-A as compared to ABLUP (as all SEP values were below the 45° reference lines (Fig. 2; GBLUP-A vs. ABLUP), confirming the superiority of the GBLUP-A model. For this reason, we used the GBLUP-A model as a reference for the extended models’ comparisons. GBLUP-AD and GBLUP-ADE were proven to be the best models for HT and WD, respectively (Fig. 2; GBLUP-AD vs. GBLUP-A (left panel) and GBLUP-ADE vs. GBLUP-A (right panel) for HT and WD, respectively).

Generally, the predictive accuracy of random folding was much higher than that of family folding, demonstrating the role of relatedness on predictive accuracy overestimation (HT 0.563–0.690 vs. 0.089–0.271; WD 0.544–0.699 vs. 0.001–0.220) (Table 2). Random folding cross-validation prediction accuracy was the lowest for ABLUP for both traits (0.615 and 0.625 for HT and WD, respectively) compared to the GBLUP models which gave a range of 0.681 (GBLUP-AD) to 0.690 (GBLUP-A) and 0.6939 (GBLUP-ADE) to 0.698 (GBLUP-AD) for HT and WD, respectively (Table 2; diagonal values). On the other hand, the pairwise prediction accuracy between ABLUP and GBLUPs (HT 0.563 to 0.622; WD 0.544 to 0.649) was lower than between the GBLUP models themselves (HT 0.681 to 0.689; WD 0.691 to 0.698) (Table 2; off-diagonal values). When GBLUP models were used to predict ABLUP, the prediction accuracies ranged from 0.619 to 0.622 (HT) and from 0.646 to 0.649 (WD), while when the ABLUP was used to predict GBLUPs, the range was significantly lowered (from 0.563 to 0.571 and from 0.544 to 0.547, for HT and WD, respectively) (Table 2). Regarding predictability, expressed as the correlation between the predicted BVs from cross-validation (PBV-CV) and the phenotype, GBLUP-A and ABLUP showed the highest values (0.285 and 0.262 for HT and WD, respectively) (Table 2; random folding, first column).

**Table 2:** Height (HT) and wood density (WD) predictability (Pearson product-moment correlations between PBV-CV and phenotype) and prediction accuracy (Pearson product-moment correlation between PBV-CV and EBV-all) within and among models (ABLUP, GBLUP-A, GBLUP-AD, and GBLUP-ADE) using random and family folding (standard errors)

	HT					WD				
PBV-CV^1^	EBV-all^2^					EBV-all^2^				
	Phenotype	ABLUP	GBLUP-A	GBLUP-AD	GBLUP-ADE	Phenotype	ABLUP	GBLUP-A	GBLUP-AD	GBLUP-ADE
Random folding										
ABLUP	0.273 (0.004)	0.615 (0.003)	0.622 (0.003)	0.619 (0.003)	0.619 (0.003)	0.292 (0.003)	0.625 (0.002)	0.649 (0.002)	0.649 (0.002)	0.646 (0.002)
GBLUP-A	0.283 (0.002)	0.571 (0.002)	0.690 (0.002)	0.689 (0.002)	0.689 (0.002)	0.257 (0.006)	0.545 (0.005)	0.694 (0.004)	0.694 (0.004)	0.695 (0.004)
GBLUP-AD	0.276 (0.003)	0.563 (0.002)	0.682 (0.002)	0.681 (0.002)	0.681 (0.002)	0.262 (0.003)	0.547 (0.003)	0.698 (0.002)	0.698 (0.002)	0.699 (0.002)
GBLUP-ADE	0.285(0.002)	0.570 (0.002)	0.688 (0.001)	0.688 (0.001)	0.688 (0.001)	0.257 (0.004)	0.544 (0.005)	0.691 (0.005)	0.691 (0.005)	0.693 (0.004)
Family folding										
ABLUP	NA^3^	NA	NA	NA	NA	NA	NA	NA	NA	NA
GBLUP-A	0.085 (0.004)	0.089 (0.010)	0.254 (0.011)	0.257 (0.011)	0.257 (0.011)	− 0.021 (0.009)	0.019 (0.012)	0.204 (0.011)	0.205 (0.011)	0.220 (0.012)
GBLUP-AD	0.087 (0.010)	0.100 (0.014)	0.266 (0.016)	0.271 (0.017)	0.271 (0.017)	− 0.036 (0.012)	0.010 (0.014)	0.189 (0.015)	0.191 (0.015)	0.207 (0.015)
GBLUP-ADE	0.084 (0.009)	0.093 (0.014)	0.255 (0.015)	0.258 (0.015)	0.258 (0.015)	− 0.038 (0.008)	0.001 (0.010)	0.184 (0.010)	0.186 (0.009)	0.202 (0.009)

^1^PBV-CV: predicted breeding values using cross-validation

^2^EBV-all: estimated breeding values using all data

^3^NA: predicted individual additive breeding value is equal to the overall mean of the model

For family folding, as indicated above, the predictability and prediction accuracy were generally much lower as compared to random folding (Table 2). The use of the ABLUP model for individual breeding value prediction for members of new families is not applicable as the relatedness is equal to zero, and the predicted value will be simply the overall mean of the model (Table 2). It is worth mentioning that while family folding produced the lowest predictive accuracy, family folding results represent the most reliable prediction as the number of independently segregating chromosome segments is maximized as opposed to the increased similarity in the random folding scenarios.

## Discussion

Tree improvement programs depend mainly on phenotypic selection and the pedigree-based average numerator relationship (***A***-matrix) for estimating genetic parameters and variance components; thus, the degree of genetic advance is dependent on the accuracy and precision of these parameters, specifically heritability and individuals’ breeding values. The utilized mating design determines mainly which genetic component can be generated (additive and dominance), and in some cases, additional efforts such as combining full-sib families with replicated clonal trials are required to estimate epistatic genetic variance (Foster and Shaw 1988; Bradshaw and Foster 1992). While OP family testing represents the most efficient method for screening large numbers of individuals in terms of cost, time, genetic sampling, and increased selection differential potential, however, OP testing suffers from inflated additive variance estimates due to the impossibility of meeting the commonly assumed half-sib structure (Namkoong 1966; Squillace 1974; Askew and El-Kassaby 1994). The availability and affordability of DNA high-throughput fingerprinting methods, such as GBS, made it possible to use SNPs to estimate the realized relationship matrix (***G***-matrix) among individuals, irrespective of their genealogy, and substitute the ***A***-matrix in estimating genetic variance components particularly in the population of forest trees (Denis and Bouvet 2013; Zapata-Valenzuela et al. 2013; Klápště et al. 2014; Muñoz et al. 2014; de Almeida Filho et al. 2016; Gamal El-Dien et al. 2016). These studies illustrated the superiority of the GBLUP and resulted in generating more precise genetic parameters, mainly due to the method’s efficiency in separating the additive from nonadditive (dominance and epistasis) genetic variances as well as accounting for the Mendelian sampling within families (VanRaden 2008; Hayes et al. 2009). In a previous study conducted to parse out additive and nonadditive genetic variances, Gamal El-Dien et al. (2016) used data from a single-environment white spruce trial and demonstrated the presence of nonsignificant dominance as well as significant epistatic genetic variances; however, the study may have produced biased estimates, because the genotype × environment interaction (G × E) component cannot be assessed in a single site. Here, we extended the single-site to a multisite model to partition the genetic variance while accounting for the G × E, using an Interior spruce OP testing population growing in British Columbia, Canada. Including the G × E in the present study has increased the model fit as demonstrated by the *R*^2^ term (variance explained by the model) and the fitted line plots (Table 1; Fig. 2) and resulted in more reliable heritability estimates. It should be pointed out that the percent variance component accounting for G × E amounted to 32.68 and 15.07% for HT (GBLUP-AD) and WD (GBLUP-ADE), respectively. These variance component percentages would have been confounded in either the additive and/or residual variances leading to inaccurate heritabilities and individuals’ breeding value estimates and ultimately affecting the estimated genetic gain (see below). Unlike animal improvement programs, where the impact of environment is managed and minimized, measuring the G × E in plant improvement is essential as markers’ effect could vary across the environment (Crossa et al. 2010).

Predictably, the results from the present study produced different additive variance estimates across the tested models (ABLUP vs. GBLUPs) (Table 1). The three GBLUP models produced lower additive genetic variance than the ABLUP model, and results concur with those reported for the single-site (Gamal El-Dien et al. 2016) and other forest tree studies (Denis and Bouvet 2013; Zapata-Valenzuela et al. 2013; Klápště et al. 2014; Muñoz et al. 2014; de Almeida Filho et al. 2016), confirming the half-sib families unfulfilled assumption. The reduced additive genetic variance subsequently lowered the heritability estimates; however, this observed reduction in the present study was smaller than the one observed in the single-site study (Gamal El-Dien et al. 2016), highlighting the benefits of using the multisite approach in producing realistic estimates (i.e., G × E inclusion). Notwithstanding the better *R*^2^ and fitted line plot of the ABLUP model (Table 1; Fig. 1) compared to GBLUP-A, the obtained precise genetic variance and breeding value (Fig. 2) estimates from the GBLUP-A demonstrate the added value of the realized relationship-based models as their estimates are devoid of hidden relatedness inflating additive genetic variance and unaccounting the Mendelian term (VanRaden 2008; Hayes et al. 2009; Gamal El-Dien et al. 2016).

The GBLUP-AD model produced surprising results with a significant dominance variance for HT relative to the additive variance with a higher *R*^2^ value supporting better model fit (Table 1). This was also illustrated by the fitted line plot and the breeding values’ SEPs graph (GBLUP-AD: Figs. 1 and 2 left panels). This trend was not observed for WD as the dominance variance was not significant (based on SE) and only accounted for a small amount of the total genetic variance, perhaps reflecting different genetic architecture between the two traits (Table 1 and Figs. 1 and 2, right panels). Similar observations were reported for Douglas-fir and white spruce (El-Kassaby and Park 1993; Gamal El-Dien et al. 2016). The significant dominance genetic variance for HT in Interior spruce mirrored that reported for loblolly pine (Muñoz et al. 2014; de Almeida Filho et al. 2016, but see Gamal El-Dien et al. 2016). Additionally, the ability to detect dominance variance is also dependent on the nature of the population and the type of markers used to construct the dominance (fraternity) relationship matrix. In a simulation study, García-Cortés et al. (2014) reported that the presence of multiallelic markers is a prerequisite for the precise estimation of the dominance coefficients, a condition, which can potentially affect the ability to estimate the dominance variance component when using exclusively biallelic markers such as SNPs. It is interesting that the HT additive genetic variance and heritability estimates did not change between GBLUP-A and GBLUP-AD, which means that the additive variance was accurately estimated in the GBLUP-A model and was not confounded with the dominance effect for this trait. Probably, this is the reason why the prediction accuracy of GBLUP-AD did not improve when compared with GBLUP-A (Table 2; diagonal).

The full model (GBLUP-ADE), which was extended to include first-order interactions, gave exactly the same results as GBLUP-AD for HT indicating the absence of all kinds of epistatic interactions; this model subsequently showed no improvement in all goodness-of-fit measures and precision estimates (Table 1, Figs. 1 and 2). These results were distinct from Gamal El-Dien et al. (2016), where HT showed significant additive × additive interaction and nonsignificant dominance, while here, HT showed a significant dominance component which was extracted from the residual variance without any effect on the additive variance. The hybrid nature of Interior spruce (*P. glauca* x *P. engelmannii*) in British Columbia (De La Torre et al. 2014) can explain such distinct results as hybridization is reflected in higher diversity and higher heterozygosity which may make the dominance effect pronounced, and additionally, dominance variance is also known to be population specific (Falconer et al. 1996). For the WD trait, however, GBLUP-ADE resulted in improved genetic variance partitioning and showed a relatively larger additive × additive component that was extracted mainly from the residual variance and to some extent from the additive variance component (Table 1), supporting the theoretical expectation that additive × additive variance is absorbed by additive and residual variances (Jannink 2007). The superiority of GBLUP-ADE model for WD was supported by the *R*^2^ estimates (Table 1), the fitted line plot, and the SEP graph (Figs. 1 and 2). A significant additive × additive component was also observed in the white spruce OP study of Gamal El-Dien et al. (2016) and in loblolly pine full-sib population (Muñoz et al. 2014). Thus, the WD results were consistent with Gamal El-Dien et al. (2016) and the present study as both studies showed nonsignificant dominance variance in addition to a significant additive × additive interaction that was extracted from the additive and residual variances. Also in both studies, substantial epistasis was detected in the genetic architecture of WD in spruce, and therefore, this result cannot be an artifact based on the population sampling and/or genotyping methodology as the two studies used completely different genotyping platforms.

The advantage of GBLUP models is their use of the realized genomic relationship among individuals regardless of their genealogy, while the ABLUP is mainly dependent on the pedigree-structure created by mating designs. Additionally, to capture the additive relatedness among individuals, the realized genomic relationship matrix is also capturing the linkage disequilibrium (LD) between the SNPs and quantitative trait loci (QTLs) and their co-segregation (Habier et al. 2007, 2010, 2013). These factors, collectively, affect the accuracy of the genomic estimated BVs (Habier et al. 2013). Most tree improvement breeding programs are in their early stage of tree domestication and suffer from their shallow and simple pedigrees which make ABLUP’s estimates somewhat questionable. Our cross-validation results support this notion as the GBLUP models produced higher prediction accuracy than the ABLUP (Table 2). Furthermore, using the GBLUPs to predict ABLUP produced better results than the reverse scenario. This is expected as the GBLUP models are capable of capturing contemporary as well as historical relatedness (Table 2; see the off-diagonal estimates). The GBLUP models’ superiority was already illustrated by Muñoz et al. (2014). In their study on loblolly pine, Muñoz et al. successfully estimated the epistatic genetic variance from a full-sib mating design with clonally replicated trials using the GBLUP approach, while the ABLUP failed to estimate the epistatic genetic variance despite having full-sib families and clonal replications.

It is noteworthy to mention that extending the GBLUP models to include the dominance (GBLUP-AD in the case of HT) and dominance as well epistasis variances (GBLUP-ADE in the case of WD) resulted in improving the breeding values’ estimates precision (Fig. 2); however, these adjustments did not improve the prediction accuracy in the cross-validation compared to the GBLUP-A (Table 2; diagonal). Such a scenario was also observed in a similar genetic variance decomposition study in the context of genomic selection for milk production in cattle (Ertl et al. 2014). This discrepancy can be explained by the fact that both dominance and epistatic genetic variances were mainly extracted from the residual term, thus resulting in no or minimal impact on the additive variance component.

The observed predictive accuracy differences between the random (retaining genetic similarity) and family (excluding genetic similarity) folding are of great importance (Table 2). Our results and those obtained from previous studies conducted on forest trees (Resende et al. 2012; Beaulieu et al. 2014a, b; Gamal El-Dien et al. 2016) clearly demonstrated consistent predictive accuracy overestimation, driven mainly by relatedness, results meeting theoretical expectations (Daetwyler et al. 2013). This situation calls for caution when genomic selection is implemented, and therefore, using family folding (removed genetic similarity between the training and validation populations) is prudent even with lower predictive accuracy for obtaining realistic gain estimates as it is solely based on the short-range LD, the backbone of genomic selection paradigm (Meuwissen et al. 2001).

**Fig. 3:**
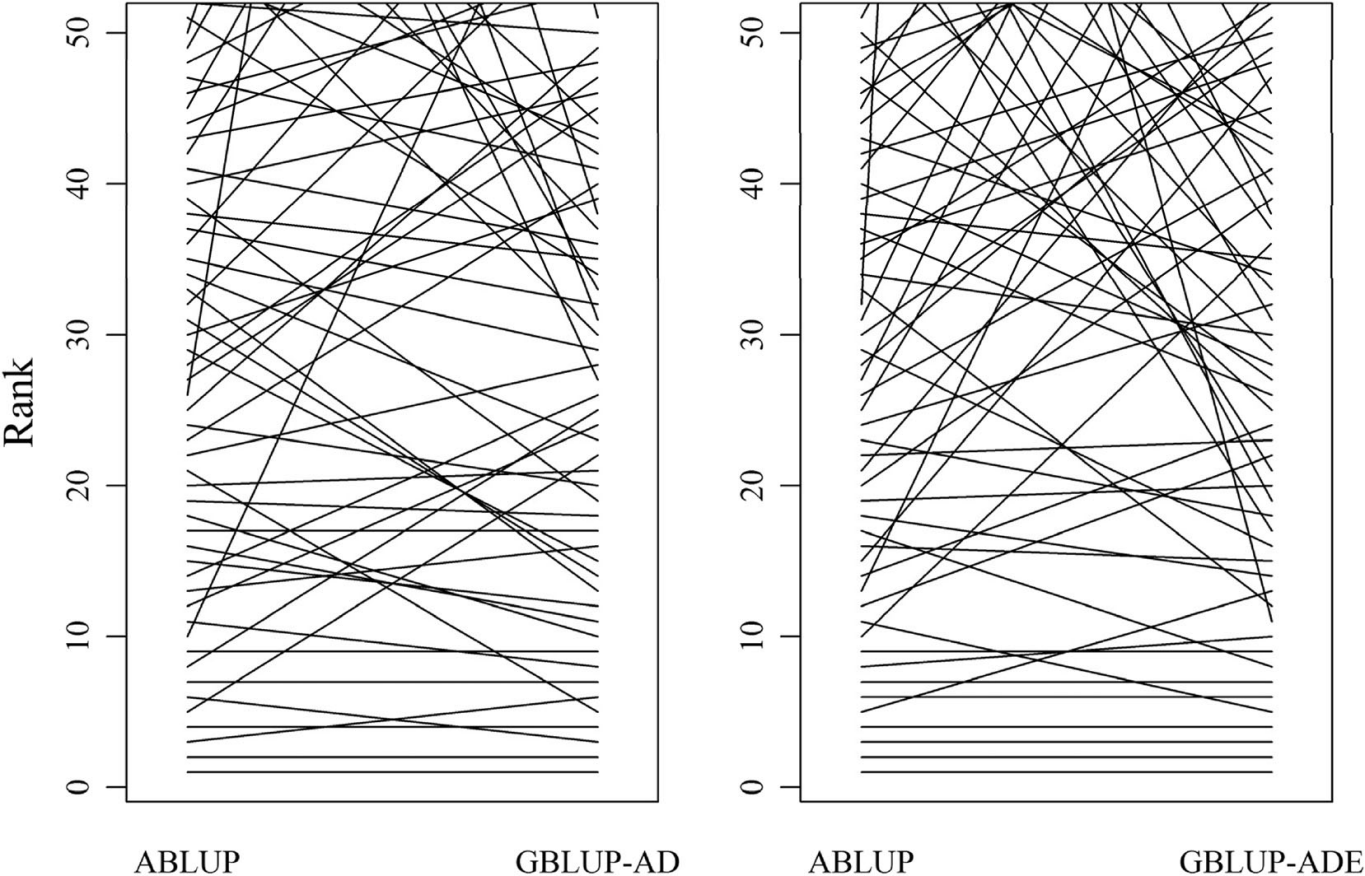
Height (left) and wood density (right) breeding value ranking plots comparing ABLUP vs. GBLUP-ADE assessments for forward selection of the top 50 performing individuals

The reported multisite genetic variance decomposition along with the selection of the best model for each studied trait is expected to improve the genetic variance partition (see above) as well as the individuals’ breeding values. We compared the ranking of the top 50 individuals for HT and WD between the pedigree-based ABLUP and the genomic-based GBLUP models (GBLUP-AD and GBLUP-ADE, for HT and WD, respectively) (Fig. 3). Only 78 and 76%, respectively, of the top 50 individuals persisted between the ABLUP (HT) and the GBLUP-AD (HT), and between the ABLUP (WD) and GBLUP-ADE (WD), both rankings indicated that some of the top ranked individuals from ABLUP have completely dropped out, suggesting the need for revaluating the expected genetic advance from the traditional ABLUP approach. Using the 1000 studied trees as a based population, we compared the ABLUP’s and GBLUP’s (HT: GBLUP-AD; WD: GBLUP-ADE) response to selection (*R* = *h*^2^ *S*; Falconer et al. 1996) estimates using each model’s breeding values and heritabilities, and truncating the population to include the top ranked 50 individuals (selection intensity of 5%), we observed 35 and 63% response to selection reduction for HT and WD, respectively. This substantial reduction in the response to selection is the product of (1) inflated ABLUP heritability estimates, (2) revised GBLUP breeding values, and (3) rank change among the top 50 individuals, and all act in concert affecting the anticipated genetic advance. This example clearly illustrates the inaccuracies of the ABLUP evaluation and also highlights the benefits of GBLUP implementation and its advantages in advancing the quick, simple, and inexpensive OP family to the forefront of forest tree progeny testing.

## Conclusions

The utilization of the genomic-based relationship in tree improvement testing and evaluation programs is implemented, and its usefulness, in particular for the simplest OP testing mode, is demonstrated. The proposed approach of genetic variance decomposition was illustrated, and additional estimates such as dominance and epistatic genetic variances as well as their interactions with the environment were obtained. The incorporation of genomic information in the analysis provided more accurate genetic variance estimates, particularly for those that could not be derived using the traditional OP pedigree-based analysis. The negative role of genetic similarity between training and validation populations on the predictive accuracy of genomic selection as well as examples of the unrealistic genetic gain estimates derived from pedigree-based analyses was illustrated. Finally, extending the GBLUP model to include multisite makes the analysis applicable to existing OP testing programs.
